# Untypical Contrast Normalization Explains the “Weak Outnumber Strong” Numerosity Illusion

**DOI:** 10.3389/fnhum.2022.923072

**Published:** 2022-07-19

**Authors:** Quan Lei, Adam Reeves

**Affiliations:** ^1^Department of Psychology, Wichita State University, Wichita, KS, United States; ^2^Department of Psychology, Northeastern University, Boston, MA, United States

**Keywords:** numerosity perception, contrast, segregation, illusion, model, contrast-dependent numerosity illusion

## Abstract

Less salient, lower contrast disks appear to be more numerous than more salient, higher contrast disks when intermingled in equal numbers into the same display (Lei and Reeves, 2018), but they are equal in perceived numerosity when segregated into different displays. Comparative judgements indicate that the apparent numerosity of the lower contrast disks is unaffected by being intermingled with high contrast disks, whereas the high contrast disks are *reduced* in numerosity by being intermingled with the low contrast ones (Lei and Reeves, 2018). Here, we report that this illusion also occurs for absolute judgements of the numerosities of displays of from 20 to 80 disks. A model based on luminance-difference contrast normalization (LDCN) explains the illusory loss of high-contrast (salient) items along with veridical perception of the low-contrast ones. The model correctly predicts that perceived numerosity is linearly related to the square-root of the number of disks, with the extent of the illusion depending on an attentionally-weighted function of contrast and assimilation.

## Introduction

The apparent numerosity of a set of items has been a topic in psychology since Jevons (1871) reported that his error in estimating the number N of black beans thrown down at random equaled 0.11(N-4.5), for 1 < N <15. Later work showed that the numerosity of a small set of identical items can be ascertained directly by subitizing (Kaufman et al., 1949; Srebro and Mandler, 1982), by rapid estimation (Jevons, 1871), by grouping, or by serial enumeration (Liss and Reeves, 1983; Trick and Pylyshyn, 1994). The numerosity of larger sets can only be estimated, however, being hard to group and laborious to count (Messenger, 1903). This paper is about large-set estimation, and an illusion from which it can suffer.

Estimation proceeds at a glance (Ross and Burr, 2010; Cicchini et al., 2016), and estimated numerosity is monotonic with the actual number (*N*) of items. Large-set numerosity is likely to be a fundamental visual attribute, being available to 6-month old infants (Xu et al., 2004), and being orthogonal to other perceptual attributes (Dakin et al., 2011; Dehaene, 2011). Thus, well-segregated displays with equal numbers of randomly-positioned elements appear equally numerous despite variations in the shape, size, location, and contrast of the elements. For example, when we (Lei, 2015; Reeves and Lei, 2016; Lei and Reeves, 2018) presented a standard display of 50 small randomly-located disks in a 10° square patch next to a similar comparison display with a variable number of disks, the point of subjective equality (PSE) was also 50 disks, even when the two displays differed in contrast. This result illustrates the constancy of numerosity over variations in contrast, or “*contrast constancy*” for short.

**Figure 1:**
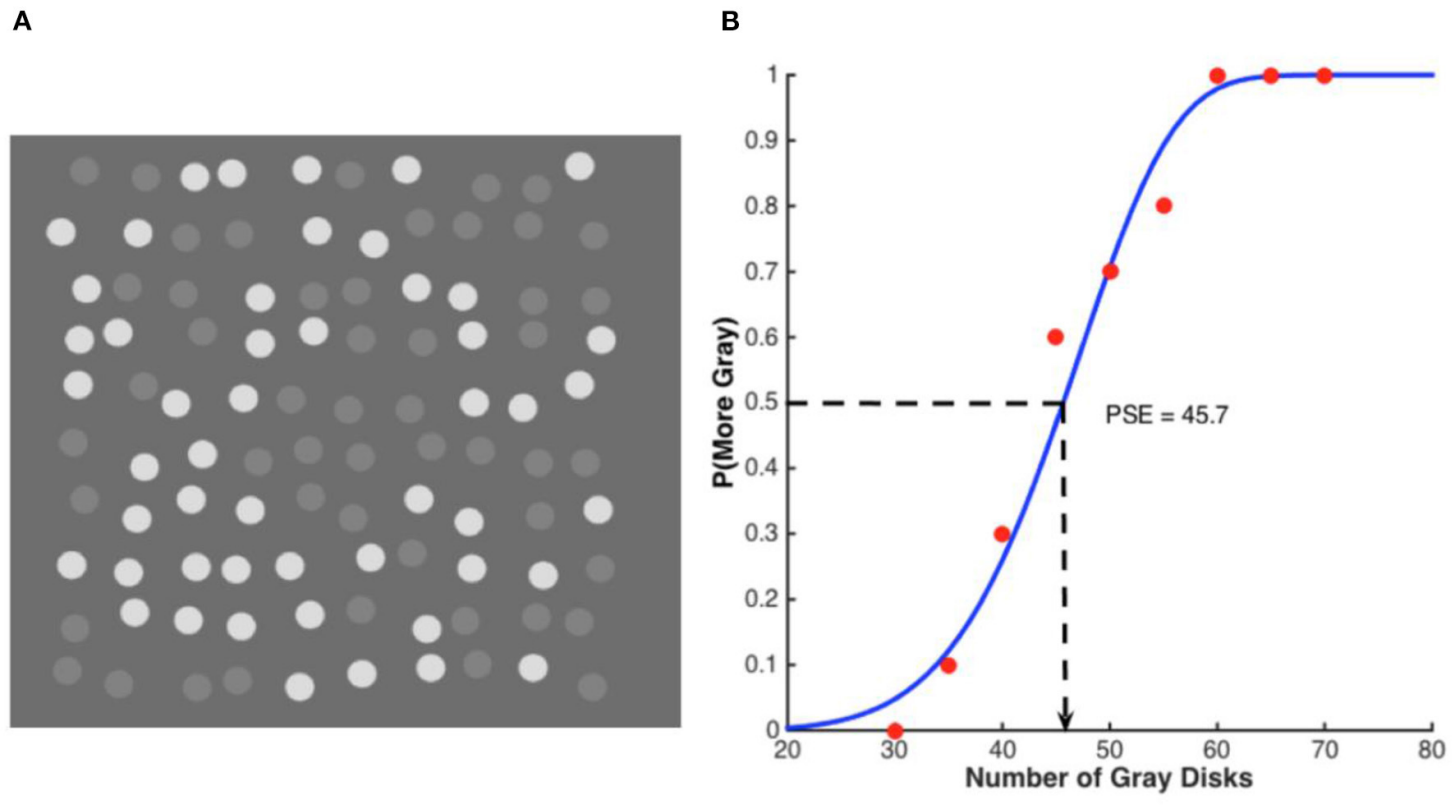
Example stimulus and psychometric function. **(A)** An example disk array in a discrimination task. The standard disk set (here, the white ones) had a fixed numerosity of 50 while the numerosity of the comparison disk set (here, gray) varied in a range of 30–70. In this example, both sets had a numerosity of 50. **(B)** An example psychometric function from one subject plots the probability of the gray disks being chosen as more numerous against their physical numerosity. The dots represent one subject's data and the curve the best-fitting Weibull function. The PSE, 45.7, indicates how many gray disks matched 50 white disks in perceived numerosity, implying that the white disks were under-estimated relative to the gray ones.

However, when the two displays were *intermingled*, such that there were now 100 disks, 50 of higher and 50 of lower contrast, the higher-contrast (white) disks appeared less numerous (mean PSE of 42) whereas the lower-contrast (gray) disks were unaffected (mean PSE of 51, not significantly different from 50). The illusion that the high-contrast elements appear less numerous, even when they are equal in number, is illustrated in Figure 1A, reprinted from Lei and Reeves (2018). We termed this illusion “the weak conquer the strong.” It is curious since one might intuit that, if anything, the weaker stimuli would be depressed toward threshold by the stronger ones, and be more likely to disappear.

The PSE's were interpolated from psychometric functions illustrated for a typical subject in Figure 1B, which shows the probability of reporting that the gray disks were more numerous than the 50 white disks, plotted against the number of gray disks. The PSE was 45.7 and the inter-quartile range, 14. The steepness of the psychometric function is typical and indicates the precision with which the numerosities of the gray disks can be judged, even when intermingled with the white ones. Switching the roles of the disks so that the whites became the comparison did not affect the PSE or the precision (Lei and Reeves, 2018). The white and gray disks were easy to distinguish; had their contrasts been more similar, the disk sets could be confused with each other and the function would flatten, but in our displays, the precision for intermingled disks equaled that for segregated disks.

**Figure 2:**
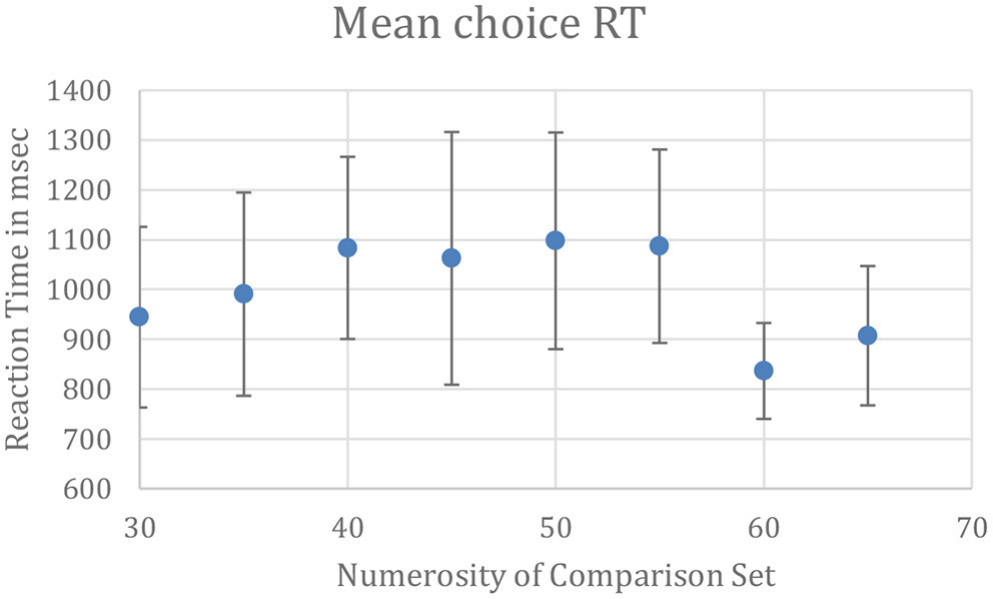
Latencies for reporting which set of intermingled disks, white or gray, were more numerous. Mean RTs were in the range of 0.8–1.2 s, typical for estimation and far faster than counting. The standard set contained 50 disks. Bars show **±**1 SE calculated between subjects; confidence intervals are 28% larger than the SEs.

The numerosity estimations were rapid, indicating that the subjects did not laboriously count the disks even when intermingled with disks of a different contrast. In an unpublished control experiment (Lei, 2015), trials were run in the same manner as in Lei and Reeves (2018), beginning with a 1 s central fixation followed by the disk display for 1.5 s, which then turned blank. Subjects pressed one key when they perceived more gray than white disks and pressed a different key for the reverse. The standard set contained 50 disks and the comparison set, 30 < *N* < 70 disks. Mean reaction times (RTs) for 8 subjects are plotted as a function of *N* in Figure 2. RTs were slowest when the comparison also contained around 50 disks, making the choice more difficult, but in all cases, mean RTs were in the range of 0.8–1.2 s, similar to RTs reported earlier for estimation and much faster than counting (Liss and Reeves, 1983).

In this paper, the illusion that white disks are fewer than an equal number of gray disks is referred to as the “numerosity illusion” throughout. The purpose of the paper is to study the numerosity illusion over a wide range, namely, from *N* = 20–80 disks. An *absolute* judgement method was used, in which subjects reported the numerosity of one set, white or gray, while ignoring the other set, in contrast to the *comparative* method (reporting which set was more numerous) used by Lei and Reeves (2018) and illustrated in Figure 1. Displays were either segregated, with contrast constancy anticipated (e.g., Dakin et al., 2011), or displays were intermingled, with the numerosity illusion expected to develop (Lei and Reeves, 2018).

Three different intermingled conditions were included to test a possible role for selective attention. Hypothetically, the need to compare the intermingled disk sets in Lei and Reeves (2018) might have generated the numerosity illusion because the weaker (gray) disks required more attention to be enumerated, leaving the white disks less attended—so apparently less numerous. Intermingled displays were therefore either *pre-cued*, so that the subject could attend to the disks to be reported and ignore the rest, or *post-cued*, in which case they had to attend to and retain both sets of disks in short-term visual memory until the cue to report. Pre-cued and post-cued trials were compared to *blocked* trials, in which the subject could attend to the same set of disks, white or gray, throughout a block of trials.

Sets of randomly-positioned identical disks were presented in a fixed 10° square area to avoid providing visual cues to segmentation (Franconeri et al., 2009), connectedness (He et al., 2009), spatial structure (Ginsburg, 1991), size (Ginsburg and Nicholls, 1988), or total area (Hurewitz et al., 2006), all of which can affect numerosity, and would therefore need randomizing with respect to the variable of interest here, namely, contrast, should they be introduced into the experimental paradigm. Our displays were purposefully plain.

## General Method

### Subjects

From 6 to 8 different undergraduates, all with 20/20 vision or better, participated in each condition. Subjects gave informed consent before the experiment and were permitted to leave at any time, although none did. They were told that they would evaluate the number of randomly-shown disks that were presented to them under various conditions of brightness and contrast. The protocol was approved by the institutional review board (IRB) of Northeastern University.

### Stimuli

Stimuli were generated using Matlab in conjunction with Psychtoolbox (Brainard, 1997) and presented on a Dell LCD monitor viewed at 60 cm. The stimuli consisted of disks (0.5° across) distributed pseudo-randomly over a square patch of 10° on each side (as in Figure 1A). The patch was divided into a 12 by 12 grid of imaginary cells, and the disks were placed in a subset of these cells with jittering. In *intermingled* displays, two sets of disks with different contrasts were intermingled in a single patch presented at the center of the screen. In *segregated* displays, only one set of disks was presented, also in the same 10° square patch. Since the disks were small relative to the field, *disk contrasts* were defined in Weberian rather than in Michelson terms (Peli, 1996) as δL/L, where L is the background luminance and δL is the luminance difference between the disk and L. White (74.0 cd/m^2^) and gray (26.1 cd/m^2^) disks were presented on a dark gray field of 18.2 cd/m^2^. Thus, both sets of disks had positive contrasts, these being 3.11 (white) and 0.43 (gray).

The *set size* (*N*) is the number of disks in the target (comparison) set, that is, the set to be reported. *N* was always randomized over trials, and varied from 30 to 70 when blocked or from 20 to 80 otherwise. Thus, the total number of disks in intermingled displays was *N* + 50 disks, as the standard set contained 50 disks.

### Procedure

There were two main conditions, *segregated* and *intermingled*. In the *segregated* case, the disk display in each trial comprised either gray or white disks. The set size, *N*, varied randomly from *N* = 20–80 in steps of 10. Set size and target color (gray or white) were both randomized across trials, so subjects did not know which to expect. In the *intermingled blocked* condition, gray and white disks were intermingled. Trials with gray disks as targets and trials with white disks as targets were run in separate blocks of 45 trials. Four of the 8 subjects completed two gray trial blocks first and then two white blocks, while the other four were run in the opposite order. Set size, *N*, was (untypically) randomized from *N* = 30–70 in steps of 5.

In the *intermingled randomized* condition, subjects were pre-cued (8 subjects) or post-cued (7 other subjects) to judge the number of either the gray disks or the white disks in each intermingled display. The target set size (the number of disks to be judged) varied from *N* = 20–80 in steps of 10. There were always 50 disks of the other color to be ignored, so if the targets were gray, then *N* gray disks were intermingled with 50 white ones, and if the targets were white, then *N* white disks were intermingled with 50 gray ones. Whether the target set was gray or white was randomized across trials.

**Figure 3:**
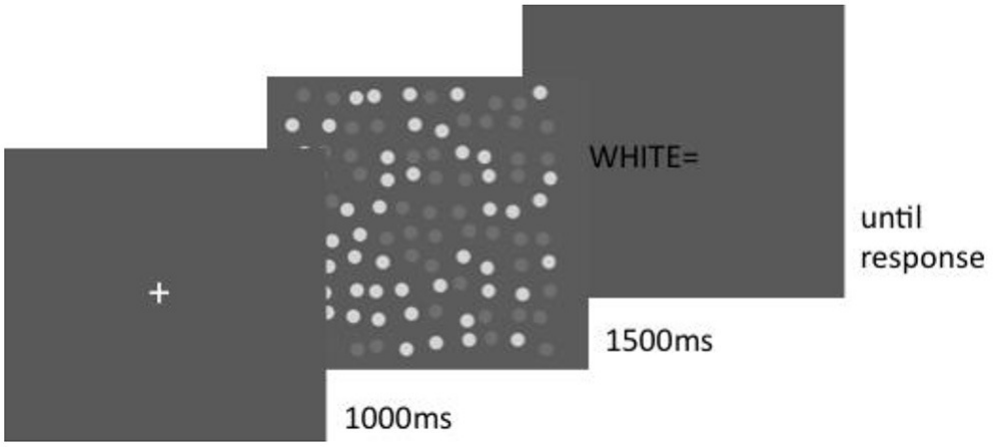
Post-cue intermingled display. A verbal prompt as to which disk set to enumerate was given after the display until the subject entered his or her response.

A *post-cue* intermingled display with white target is illustrated in Figure 3. In a *pre-cue* trial, the fixation cross would have turned white (or gray) for 1 s before the display. Timing was otherwise the same in all conditions; a fixation cross was presented for 1 s, followed by a disk display for 1.5 s. Subjects input their numerosity estimate after each trial by using the keys on a standard number pad. The inter-trial interval was 3 s.

### Task

Subjects were told to evaluate the numerosity of the target set while ignoring the non-target set. They were not told the other (non-target) set in the intermingled display had a fixed numerosity (50).

### Design

Set size and target disk color were within-subject variables. Each set size (*N*) was repeated 10 times for each combination of *N* and two disk colors (white or gray), for each subject. Thus, subjects each ran 140 trials when the step size was 10 and 180 trials when the step size was 5. The trials were divided into four blocks, with 35 or 45 trials in each block. Subjects could rest between blocks. They completed 10 practice trials without feedback for each disk color, to become familiar with the procedure. Subjects were then run for about 20 min. in the main experiment, with short breaks between blocks. Each trial lasted 6–7 s.

## Results

### Segregated Displays

**Figure 4:**
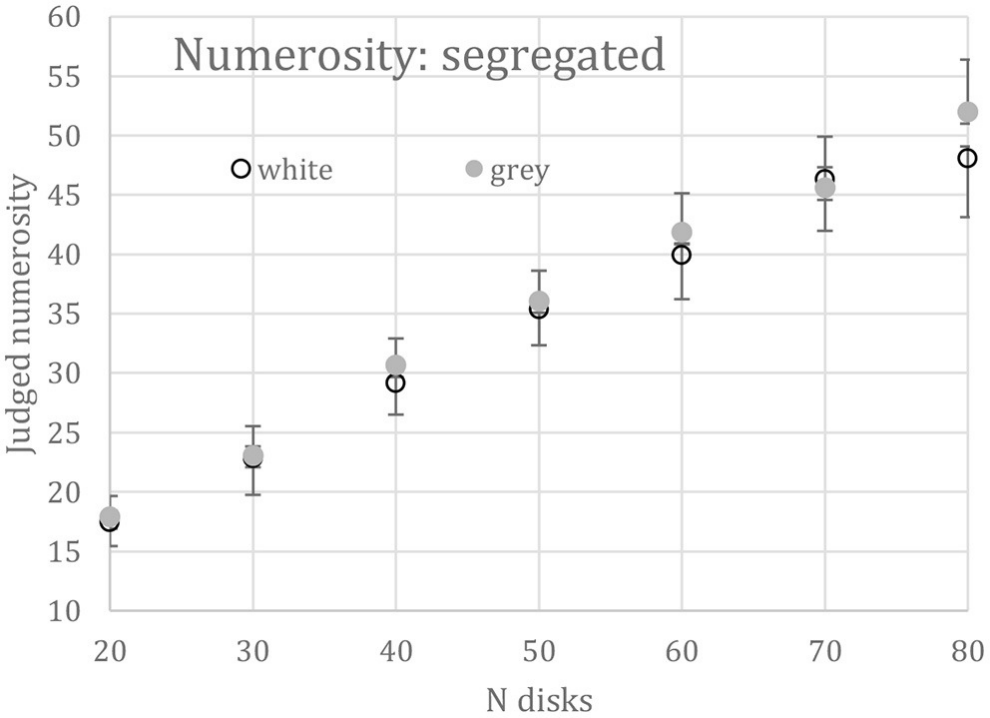
Mean numerosity of segregated displays of 20 to 80 all white or all gray disks. Bars show +1 between-subjects SEs for the gray disks and −1 for the white ones; confidence intervals are 28% larger than the SEs.

A 2 (disk color) × 7 (disk number) two-way repeated-measures ANOVA was performed on the mean numerosity estimates from 6 subjects. Judged numerosity increased significantly [F_(6, 30)_ = 68.59, *p* < 0.01] over the 7 set sizes (*N*), as shown in Figure 4. As predicted by contrast constancy, the gray disks were no more numerous than white ones, the effect of disk color being insignificant [*F*_(1,5)_ = 3.73, *p* = 0.11]. There is a hint in Figure 4 that 80 gray disks appeared more numerous than 80 white ones, but the color-number interaction was not significant [*F*_(6,30)_ = 1.18, *p* = 0.34].

### Intermingled Displays: Pre-cue

**Figure 5:**
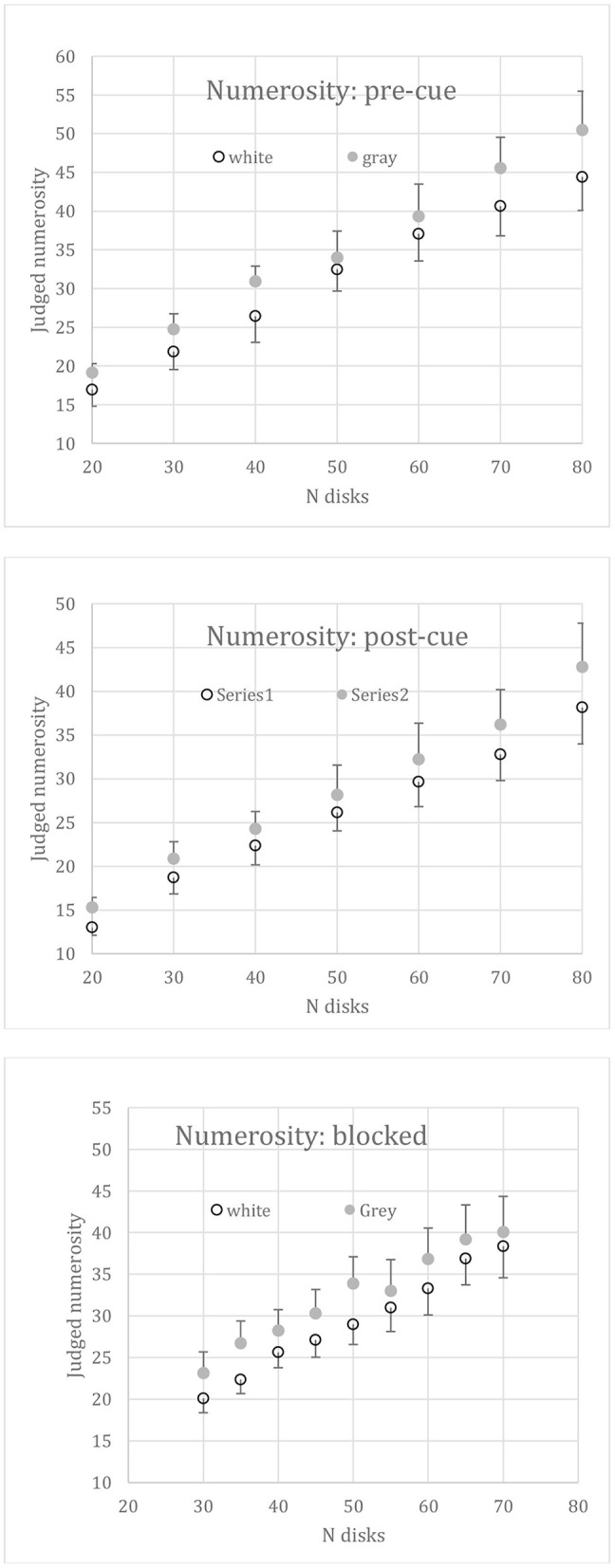
Mean judged numerosity vs. set size (N) when disk sets were intermingled. i.e., both white and gray disks appeared together on each trial. Top: the target set (i.e., the set to be judged) was randomized but pre-cued. Middle: the target set was randomized but post-cued. Bottom: target set was blocked. Error bars: +1SE for gray disks, −1SE for white disks.

One of the 8 subjects run with a pre-cue was an obvious outlier and was excluded. The mean numerosity judgements of the remaining 7 subjects is plotted in Figure 5, top. Numerosity again increased with set size [*F*_(6,36)_ = 45.88, *p* < 0.01]. The numerosity of gray disks was overestimated relative to the white disks [*F*_(1,6)_ = 30.88, *p* < 0.01] equally over the whole range of set size (*N*), there being no interaction [*F*_(6,36)_ = 0.71]. (The outlier subject also overestimated the numerosity of gray disks, but to a much-exaggerated degree). Thus, the numerosity illusion persisted even when a pre-cue permitted subjects to attend fully to the target disks.

### Intermingled Displays: Post-cue

The mean numerosity judgements of 7 subjects are plotted in Figure 5, middle. Numerosity again increased significantly with set size [*F*_(6,36)_ = 64.21, *p* < 0.01]. Gray disks were judged as more numerous than white ones [*F*_(1,6)_ = 63.31, *p* < 0.01], again equally over the whole range of set sizes, there being no interaction with *N* [*F*_(6,36)_ = 0.65]. This result indicates that the illusion remains even when the subject must retain both sets and is directed to attend to one or other set only in short-term memory.

### Intermingled Displays: Blocked

The mean numerosity judgements of 8 subjects are plotted in Figure 5, bottom, against set size, *N*. Numerosity again increased over the 9 set sizes [*F*_(8,56)_ = 50.18, *p* < 0.01]. Gray disk numerosity was overestimated relative to the white disks [*F*_(1,7)_ = 6.80, *p* = 0.04], again with no interaction [*F*_(8,56)_ = 0.64].

### Summary of Numerosity

The grand mean absolute numerosities in each condition are given in Table 1, along with the standard error of the difference between gray and white disks. The mean number presented, *N*, was 50 in all conditions. The grand mean numerosity reported was 31.3. The lower overall report in the post-cue condition (27.1, compared to 32.9 in the remaining conditions) may reflect a loss in short-term memory.

**Table 1:** Mean absolute numerosity.

**Condition**	**White**	**Gray**	**Diff**	**SEdiff**	***t***
Segregated	34.15	35.31	1.15	1.21	0.95
Blocked	29.31	32.38	3.06	0.97	3.16
pre-cue	31.41	34.90	3.49	1.37	2.55
post-cue	25.83	28.54	2.71	0.96	2.83

When segregated, the difference in numerosity (“Diff” in Table 1) of 1.15 in favor of gray over white disks was not significantly different from zero. When intermingled, the mean Diffs of 3.06, 3.49, and 2.71, for the blocked, pre-cue and post-cue condition, respectively, were all significant by *t*-tests (*p* < 0.05), their grand mean of 3.08 representing an illusion magnitude of 6.16%.

## Discussion of Absolute Numerosity

A precondition for the appropriateness of our method in the intermingled condition is that disks can be selected perfectly by color, implying that any loss of disk numerosity must be due to a visual interaction, not to task difficulty. The results show that an equal number of gray disks was reported whether presented alone or intermingled with white disks and pre-cued, not only for the overall mean of 35 reported (Table 1) but at every level of *N* (compare Figure 4 with the top panel of Figure 5). Thus, the pre-cue permitted the subject to select out the cued disks from the remaining intermingled disks just as well as when the same disks were presented alone. Selecting the cued disks was not problematic for the subjects.

Given that this precondition was met, the results appear straightforward. When intermingled, the illusion persisted over all set sizes tested and occurred whether the target display was cued or not. Since the effect manifests itself in absolute judgments when no explicit comparison between disk patterns is involved, it cannot be simply attributed to any post-perceptual comparative biases, as might have occurred when judging one set of disks vs. the other (as in Figure 1A). Moreover, the fact that the illusion occurred whether cued or not rejects any explanation solely in terms of selective attention. Attention may modulate, but does not cause, the numerosity illusion.

An important issue that our method does *not* resolve concerns the overall underestimation of white and gray disks, even when segregated; on average, 35 are reported when 50 are shown. Possibly all 50 are seen veridically, but numerical magnitudes are subjected to a compressive power law at some post-perceptual stage. Alternatively, the 50 disks are grouped or otherwise visualized such that 35 are perceived and are reported as such. In this case, one might describe both sets of disks as suffering from an illusion of paucity. Other methods of magnitude estimation such as cross-modality matching would be required to sort this out. What is important here is that the function relating number to judged numerosity is the same for segregated gray and white disks; it is only when they are intermingled does the report of white ones drop. Thus, we are secure in describing the loss of intermingled white disks as a perceptual illusion of numerosity, in agreement with the comparative method of Lei and Reeves (2018), rather than as an artifact of magnitude estimation.

## Model of Contrast Normalization

As already stated, we follow those authors who have assumed that large-set numerosity is perceived through a dedicated visual mechanism (Feigenson et al., 2004; Burr and Ross, 2008; Dehaene, 2011; Anobile et al., 2014; Cicchini et al., 2016; Burr et al., 2018), the postulated mechanism likely being some form of texture processing (Morgan et al., 2014; Balas, 2016). In displays like ours with a fixed surface area and random positioning of elements within it, textures differ in *total contrast energy*, E (Durgin, 1995; Dakin et al., 2011; Gebuis and Reynvoet, 2012) rather than, say, in bounding contour or other visual cues (Miller and Baker, 1968). For discrete stimuli such as disks, E is:


(1) $$\begin{array}{l} \text{E}=\Sigma {\text{c}}_{\text{i}}^{2} \end{array}$$

where c_i_ is the contrast of the i-th element and the summation is taken over all N elements in the display. If the elements have the same contrast c, as in our displays, then E = Nc^2^ (Note that the definition of E is more complicated with continuously-varying stimuli such as Gabors: Dakin et al., 2011). That contrast polarity does not affect numerosity in segregated displays (Lei and Reeves, 2018) is explained by contrast energy, since squaring equates disks with positive contrast to those with negative contrast.

The total contrast, C, is the square-root of contrast energy; C = √E = ^c^√N. With segregated displays of equal contrasts but different numbers of elements, C varies with N and can indicate numerosity directly. However, with equal numbers but different contrasts, the total contrast of the lower contrast display, say C_lo_, will be less than that of the higher contrast display, say C_hi_. To explain contrast constancy it is essential to normalize C_lo_ and C_hi_ by their peak-trough contrasts, c_lo_ and c_hi_. Since C_lo_/c_lo_ = C_hi_/c_hi_ = √N, normalization cancels out contrast differences. Therefore judged numerosity, J, should be linearly related to √N:


(2) $$\begin{array}{l} \text{J}=\text{A}[\surd \text{N}-\text{B})] \end{array}$$

**Figure 6:**
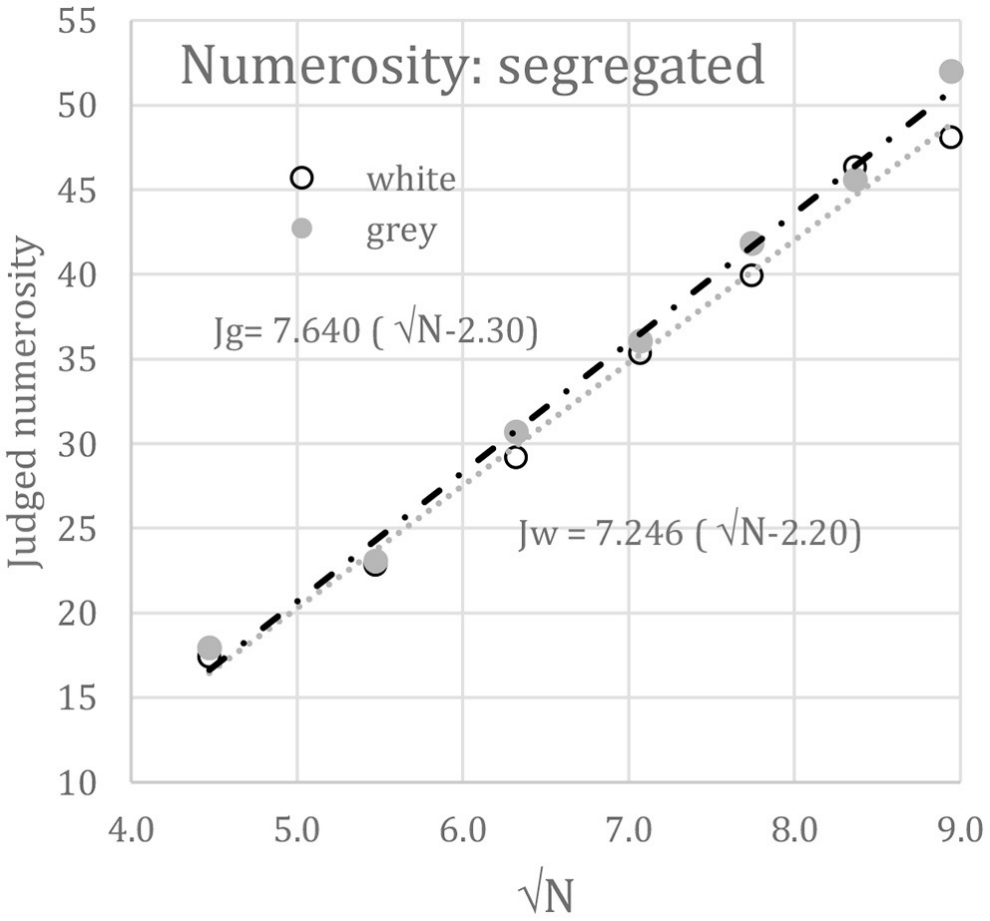
Mean judged numerosity (J) vs. √N when disk sets were *segregated*; i.e., only white disks (Jw) or only gray disks (Jg) appeared on each trial.

Here, the offset, B, acts to exclude N < 6, the region of subitization, and therefore should be about √6 or 2.4. The slope, A, acts as a scale factor. Figure 6 plots against √N the best-fits of Equation 2 to the *segregated* white (Jw) and gray (Jg) disk numerosities taken from Figure 4. Since white and gray numerosities did not differ statistically, Equation 2 was also fit to their means, for which A = 7.36 and B = 2.25 (*r* = 0.996). The fits are essentially perfect.

### Intermingled Displays

If contrast normalization worked in all cases, contrast constancy would occur in intermingled as well as segregated displays, and the numerosity illusion would not occur. However, it does occur. Lei (2015) and Lei and Reeves (2018) explained why the illusion does occur, such that the “weak conquer the strong,” by postulating that total contrast, C, depends on difference between *the disk luminance and the next lower luminance*. Such luminance differences may aid discrimination of stimuli processed by the same visual channel when no other cues exist to do so (Morgan et al., 2014).

For convenience, we refer to this *luminance-difference contrast normalization hypothesis* by an acronym, LDCN. Everything that follows is written for the normal polarity displays, to simplify symbols, but LDCN applies equally to reverse polarity displays except that the comparison is now to the next *higher* luminance.

According to LDCN, the contrasts δG/L of the weaker (gray) disks in intermingled displays (e.g., Figure 1A) are determined by the luminance difference between them and the field, L, that is, δG/L = (G-L)/L for gray disk luminance G, since the field has the next lower luminance. Thus, the LDCN contrast of the gray disks equals their Weber contrast both when the (weaker) gray disks are intermingled with (stronger) white disks and when they are segregated. Thus, gray-disk numerosity should be the same—predicting no illusion for gray disks in either normal or reversed polarity intermingled displays, as was found by Lei (2015) and Lei and Reeves (2018).

The contrasts of the white (or black) disks, when *intermingled*, however, are determined by the luminance difference between them and the *gray* ones, these having the next lower luminance. Thus, the LDCN total contrast of the white disks is less when intermingled than when segregated. However, the normalization factor, c = δW/L, does not change, as the peak-trough difference equals the luminance difference between the white disks and the field in both cases. Therefore, the normalized contrast of white intermingled disks is less than that of white segregated disks, and so numerosity constancy is violated in the observed direction (Lei and Reeves, 2018).

Denote the LDCN contrast difference by d = |δW/L - δG/L|, where δW/L is the Weber contrast (c) of the white disks and δG/L is the Weber contrast of the gray disks. Then


(3) $$\begin{array}{l} {\text{J}}_{\text{w},\text{ intermingled}}=\text{A}\left[\left(\text{d}/\text{c}\right)\text{√N}-\text{B}\right] \end{array}$$

the factor d/c dictating the size of the illusion. Absent gray disks, d = c and Equation 3 reduces to Equation 2, the equation for segregated displays.

A prediction of LDCN is that the illusion should disappear for intermingled displays of *opposite*-polarity displays (white and black disks on a gray field), energy being the same as it would be in segregated displays. However, the illusion should return if the white and light gray disks on a dark field, which both have positive polarity, are replaced by black and dark gray disks on a light field, which both have negative polarity. Indeed the extent of the illusion should be the same for negative as for positive polarity displays, since E is unaffected by polarity. Both of these predictions of LDCN held up (Lei and Reeves, 2018).

A more subtle implication of the LDCN hypothesis is that *reducing* the luminance difference between the stronger and the weaker elements in an intermingled display should *increase* the numerosity illusion, since d is proportional to the luminance difference, but c is not. Indeed, Lei and Reeves (2018) reported that the illusion in a positive contrast display increased from a 16% reduction in the numerosity of the white elements to 27% by raising the luminance of the light gray elements toward that of the white ones – while keeping them visually distinct.

Since by hypothesis the numerosity of the stronger elements is reduced by the weaker elements in the *same* display, any manner of visually segregating the weaker from the stronger elements should suffice to eliminate the illusion by providing another cue to distinguish them (Morgan et al., 2014). Experiments were therefore run in Lei (2015) to test whether segregating the displays, not only spatially or by contrast polarity, as in Lei and Reeves (2018), but also by depth, shape, density, and duration, eliminates the numerosity illusion. In brief, segregating the elements always eliminated the illusion and returned numerosity constancy, as predicted.

Lei (2015) and Lei and Reeves (2018) were able to reject several other explanations of the illusion, including misclassification (counting white disks as gray ones more often than vice-versa), occlusion (by white disks of implicit gray ones), bounding contour (gray disks seeming to enclose more space than white ones, so appearing more numerous), and adaptation (the subject adapting faster to the more salient disks, and this lowering their numerosity). The absolute numerosity data shown above also reject the pure selective attention hypothesis.

Note that the LDCN contrast of the intermingled white disks, *d* = |δW/L - δG/L|, equals the sum of the (positive) white disk Weber contrast and the (negative) gray disk Weber contrast, that is, gray disk *assimilation*. Dresp-Langley and Reeves (2012) reported both contrast and assimilation occur for isolated gray patches on a lighter or darker gray field. In an important modeling exercise, Rudd (2010) showed that disk lightness in general depends on a *weighted* difference between disk contrast and assimilation due to a surrounding ring, δW/L - wδG/L, the weight (w) depending on attention. Our displays are not of the disk/ring variety, but yet may obey Rudd's rule. In the definition of LDCN used in Equation 3, w = 1, but below we discuss fits in which w <1, that, assimilation is weighted less than contrast.

We note here a limitation of Equation 3; there is no term for adaptation. However, Grasso et al. (2022) found that the numerosity of display of 29 colored items at constant luminance was reduced by adaptation to the same color, such that 34 items were matched to 29 items, a drop of 17%, while being uninfluenced by adaptation to a different color. Critically, color assimilation was used to separate perceived from physical color, and only the former adapted numerosity. We do not know if such adaptation would alter the extent of the numerosity illusion, and if so, how Equation 3 should be modified.

## Model Fits to Absolute Numerosity Judgements

**Figure 7:**
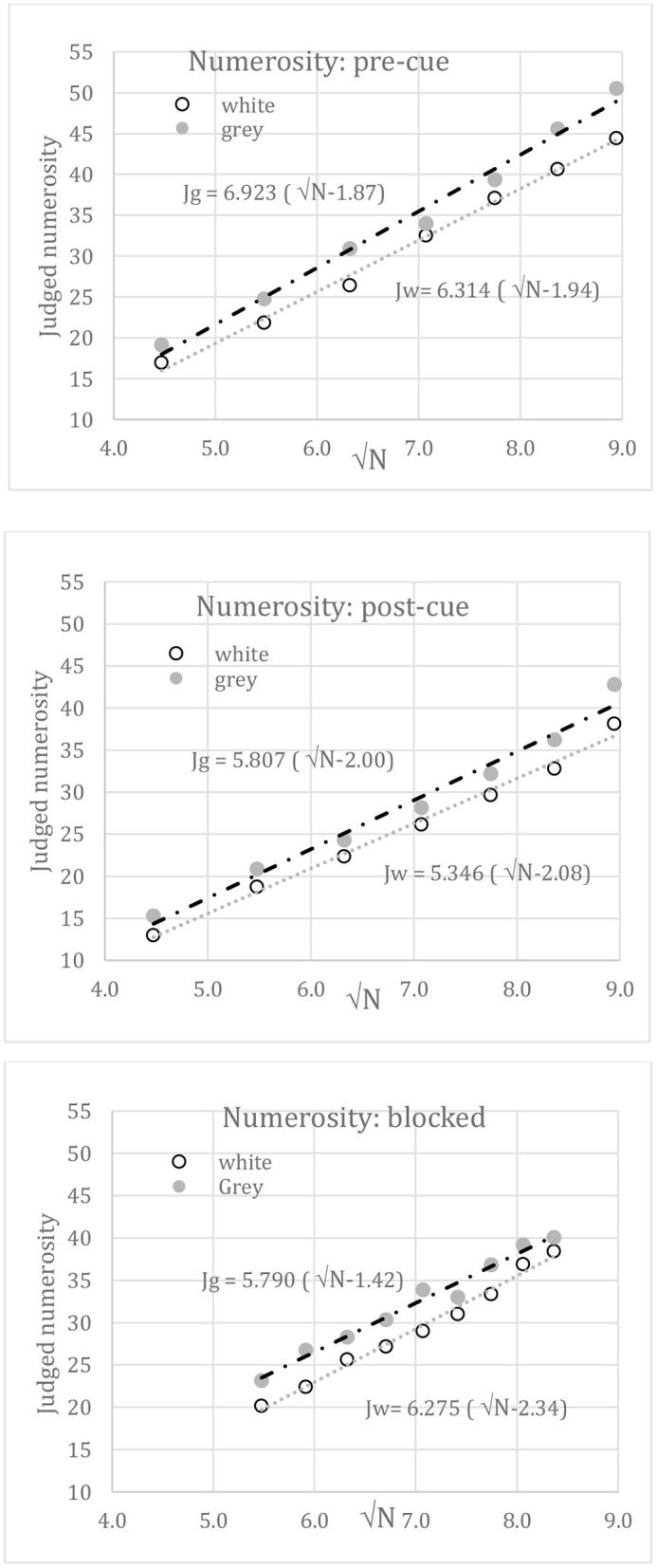
Mean judged numerosity vs. √N when disk sets were intermingled. Top: the set to be judged was randomized and pre-cued on each trial. Middle: the set to be judged was randomized and post-cued. Bottom: the set to be judged was blocked. Lines show linear best-fits to √N. These fits are empirical.

Figure 7 plots, against √N, the best-fits of Equation 2 to the *intermingled* white and gray disk numerosities taken from Figure 5 for pre-cued (top), post-cued (middle), and blocked (bottom) conditions. All correlations were 0.981 or better. Parameters A and B were free to vary (see Table 2). In these unconstrained fits, white disks may seem fewer than gray disks due to reduced slope (A) or to increased offset (B); it appears in Table 2 that both A and B vary (Note that in the segregated condition, A and B should not vary with disk color, but in fact do so due to the insignificant loss of white numerosity at N = 80).

**Table 2:** Best-fit parameters A, B to segregated or intermingled (blocked or cued) grey and white disk numerosities.

**Condition**	**A gray**	**A white**	**B gray**	**B white**
Segregated	7.64	7.25	2.30	2.20
Blocked	5.79	6.28	1.42	2.34
pre-cue	6.92	6.31	1.87	1.94
post-cue	5.81	5.35	2.00	2.08

### Discussion

The absolute numerosity judgements in both segregated and intermingled displays can be fit by linear functions of √N with high precision (r > 0.98). The grand mean offset (B) applied to √N is 2.02, suggesting subitizing below N = 5, as reported before (e.g., Liss and Reeves, 1983). This reinforces LDCN but does not exclude other possible laws, such as Weber's law (Jevons, 1871; Dehaene, 2003) or a different compressive power law (Krueger, 1972, 1984), as the range of N is limited. Indeed, the current data fit the Weber law well (*r* = 0.987). However, the fits in Figures 6, 7, and a similar fit to the pre-cued absolute numerosity estimation data of Krueger (1984), for which J = 11.4(√N-2.74), 10 < N <200, *r* = 0.992, show that a √N law is a candidate.

LDCN contrast is an untypical measure but is analogous to an idea of Peli (1996), that the perceived contrasts in each spatial frequency band are determined by the amplitudes in that band compared to the amplitudes in the next lower band (only the very lowest band being contrasted against the field). Here the sizes, densities and distributions of the white and gray disks placed them all in the same spatial frequency band, so this is only an analogy, but we mention it as an alternative to defining contrast relative to the field.

The importance of contrast energy in determining numerosity is clear in our conditions in which disks were randomly located. Once displays are structured, however, additional factors come into play. For example, connecting pairs of elements by thin lines reduces numerosity as if some pairs are bound into single units (He et al., 2009). Grouping elements can alter perceived numerosity (Ginsburg, 1976, 1991; Im et al., 2016; Poom et al., 2019), though not always (Moscoso et al., 2021). It appears that judgements of numerosity rely not only on the low-level signal provided by total contrast energy, but also, like all other percepts, on higher-level Gestalt factors such as crowding (Anobile et al., 2015) grouping and connectivity, factors which we ignore here.

### A Theoretical Basis for the Model Equation; Rudd's Rule

According to LDCN, the values of A and B obtained in the segregated displays should apply to the intermingled conditions with d/c multiplying √N. However, a fit of Equation 3 using the best-fit value of A, namely 7.00, when B was constrained to its grand mean of 2.02, badly over-predicted the illusion for pre-cued intermingled white disks when d/c was calculated based on the unweighted white and gray disk contrasts; the mean illusion, in fact 3.49, was predicted to be 6.30.

**Figure 8:**
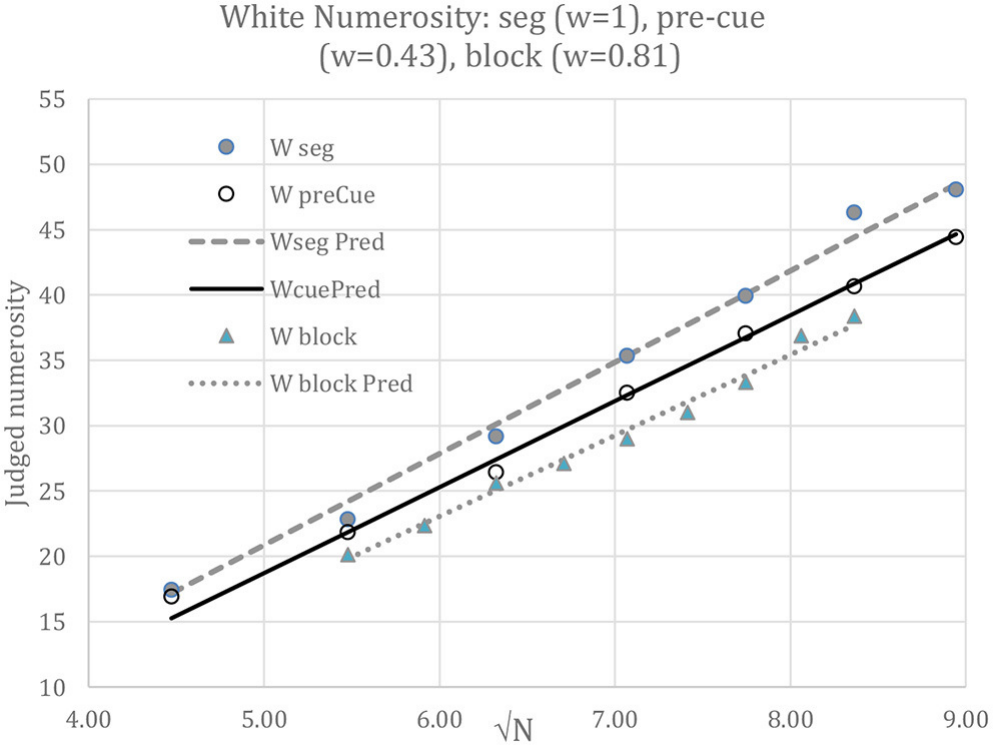
White numerosity when segregated (“seg”), as predicted by Equation 2, and when intermingled and either pre-cued or blocked, as predicted by Equation 3. Data are shown by dots and predictions by straight lines. Parameters A = 7.00, B = 2.02 were first fit to judgements of the segregated W disks. Parameters w = 0.43 and w = 0.81 were then best fit to judgements of the pre-cued and blocked intermingled W disks, with A and B unchanged. The different weights in different conditions of attention illustrate how attention can be captured in the model expressed by Equation 3. However, direct evidence that attention can alter the effective contrast energy that underpins the numerosity illusion is lacking, so this finding remains suggestive rather than conclusive; further evidence would be required to confirm it.

Therefore, Rudd's (2010) rule was applied in which the balance of contrast to assimilation is weighted by w, such that the contrast difference d, originally (W-G)/L, now equals (W-wG)/L = δW/L - wδG/L. The pre-cued intermingled white disks were least-squares best-fit with w = 0.43, so that d/c = 0.939, and the blocked intermingled white disks with w = 0.81, so that d/c = 0.885, with parameters A = 7.00 and B = 2.02 fixed. The outcome is shown in Figure 8. The fits are excellent (*r* > 0.995), but there is no independent evidence to determine the values of w, as might come from a separate assessment of the extent of assimilation. However, it is suggestive that assimilation in the blocked condition outweighed that in the pre-cue condition, as if the pre-cue permitted greater selection of the white disks and hence a reduced influence from the gray disks.

## General Discussion

The numerosity illusion presents a paradox; in almost all situations, the estimated numerosity of large sets of distinct elements is independent of other visual variables, but when salient items (disks or even bars- Lei, 2015) are intermingled with weaker items and there is no further cue to segregate them, the salient items seem reduced in number. Given that the weighted luminance-difference contrast normalization hypothesis (LDCN) explains both the effect of disk number (here) and disk contrast (Lei and Reeves, 2018) on the magnitude of the illusion, one wonders what other effects may be explained in a similar fashion. Situations in which more or less salient items are intermingled seem common in Nature, suggesting that the numerosity illusion may occur in daily life.

## Data Availability Statement

The original contributions presented in the study are included in the article/supplementary material, further inquiries can be directed to the corresponding authors.

## Ethics Statement

The studies involving human participants were reviewed and approved by Northeastern University Institutional Review Board. The patients/participants provided their written informed consent to participate in this study.

## Author Contributions

QL performed the experiments and conceived the LDCN hypothesis. AR wrote the paper and applied the analysis to the estimates of absolute numerosity. Both authors contributed to the article and approved the submitted version.

## Conflict of Interest

The authors declare that the research was conducted in the absence of any commercial or financial relationships that could be construed as a potential conflict of interest.

## Publisher's Note

All claims expressed in this article are solely those of the authors and do not necessarily represent those of their affiliated organizations, or those of the publisher, the editors and the reviewers. Any product that may be evaluated in this article, or claim that may be made by its manufacturer, is not guaranteed or endorsed by the publisher.
